# A Portable and Highly Selective Electrochemical Sensor Based on Copper–Nickel Oxide-Decorated Ordered Mesoporous Carbon for Serotonin Detection

**DOI:** 10.3390/bios16040185

**Published:** 2026-03-24

**Authors:** Thenmozhi Rajarathinam, Sivaguru Jayaraman, Jang-Hee Yoon, Seung-Cheol Chang

**Affiliations:** 1 Engineering Research Center for Color-Modulated Extra-Sensory Perception Technology, Pusan National University, Busan 46241, Republic of Korea; 2 Department of Cogno-Mechatronics Engineering, College of Nanoscience and Nanotechnology, Pusan National University, Busan 46241, Republic of Korea; sivaguru@pusan.ac.kr; 3 Yeongnam Regional Center, Korea Basic Science Institute, Busan 46742, Republic of Korea; jhyoon@kbsi.re.kr

**Keywords:** serotonin, electrochemical sensor, ordered mesoporous carbon, lower detection limit

## Abstract

Electrochemical sensors are user-friendly devices designed for the rapid and straightforward detection of target analytes. Serotonin (5-hydroxytryptamine, 5-HT) is a key neurotransmitter and neuromodulator that regulates diverse neuronal processes. Using a custom-designed screen-printed carbon electrode (SPCE) incorporating ordered mesoporous carbon–bimetal oxides of Cu and Ni (CuO–NiO–OMC), rapid and real-time detection of 5-HT was achieved. The CuO–NiO–OMC structure featured highly active CuO and NiO catalytic sites that effectively promoted the irreversible oxidation of 5-HT (vs. Ag/AgCl reference electrode). The CuO–NiO–OMC/SPCE sensor, connected to a portable potentiostat, exhibited exceptional electrocatalytic performance for the oxidation of 5-HT, with a detection limit of 42.5 nM. The sensitivity was 1.56 A M^−1^ cm^−2^, and the linear dynamic range was 0.0–80.0 µM. The CuO–NiO–OMC/SPCE sensor also demonstrated outstanding selectivity in the presence of competing neurochemicals, including norepinephrine, epinephrine, dopamine, and glutamate, as well as high concentrations of tested biomolecules and inorganic ions. Furthermore, the practicality of the sensor was demonstrated using human serum and urine samples, with recovery percentages ranging from 91.1% to 98.3%. Thus, the CuO–NiO–OMC/SPCE sensor offers an effective approach for 5-HT sensing, thereby permitting molecular-level understanding of brain function.

## 1. Introduction

Serotonin (5-hydroxytryptamine or 5-HT) is a neurotransmitter synthesized in the brain, intestine, and spinal cord and is widely distributed throughout the body. 5-HT plays a crucial role in regulating physiological functions, including sleep, mood, and endocrine activity, and its imbalance is linked to a wide range of health conditions [1]. Low 5-HT levels are associated with disorders such as depression, anxiety, migraines, unregulated hemostasis, blood clotting issues, and carcinoid syndrome [2], whereas excessively high levels can lead to 5-HT syndrome, a potentially fatal toxic state [3,4]. Because of these impacts, accurate detection of 5-HT is crucial for diagnosing various diseases and for understanding its role in neurological disorders [5]. The 5-HT levels in blood, serum, plasma, or platelets may serve as peripheral biochemical markers for these diseases, and 5-HT concentrations in blood range from 262 to 1550 nM [2]. Urinary 5-HT levels exceeding the 600 nM reference baseline are often indicative of carcinoid tumors of the gastrointestinal tract. Thus, simple, highly sensitive, and rapid detection of 5-HT in these biofluids is indispensable for the diagnosis of neurological disorders and gastrointestinal tract tumors [6].

Currently, reliable analytical methods used for the detection of 5-HT include fluorimetry [7], high-performance liquid chromatography with electrochemical detection [8], reverse injection capillary electrophoresis (CE) coupled with ultraviolet detection [9], chemiluminescence [10], enzyme immunoassays [11], ELISA, and CE–time-of-flight mass spectrometry [12,13]. These methods are cumbersome and time-consuming and often require sample pretreatment, making them impractical for rapid detection and inconvenient for routine testing. Moreover, because 5-HT is electroactive, electrochemical sensors enable fast and sensitive analysis at low cost using compact, miniaturized devices. The development of sensitive nanomaterial-modified electrodes for rapid 5-HT detection is a key research focus, driven by the need to understand how different 5-HT receptors interact within biological systems [14]. However, accurate measurement of 5-HT is challenging due to significant interference from other biological compounds, primarily because the oxidation potential of 5-HT (0.38 V vs. Ag/AgCl reference electrode) is close to that of dopamine (DA), epinephrine (EP), and norepinephrine (NE) at 0.22 V vs. a Ag/AgCl reference electrode, causing their voltammetric signals to overlap. This issue is further complicated by the presence of ascorbic acid (AA), which has a similar oxidation potential and exists at high concentrations in vivo. Compounded by the general demand for greater sensitivity, these obstacles have motivated the pursuit of faster, simpler, and more sensitive methods, leading to substantial research on nanomaterial-modified electrodes for measuring serum and urine 5-HT levels [15].

Sensitive nanomaterials accelerate electrochemical reactions by acting as heterogeneous catalysts that lower the overpotential required for 5-HT electro-oxidation. Intensive research on controlled size, shape, and three-dimensional architecture is both academically and commercially appealing because it supports the development of advanced functional materials [16]. Ordered mesoporous carbon (OMC) has emerged as an ideal support matrix because of its cost-effectiveness, three-dimensional structure, porosity, exceptional electrical conductivity, and robust structural integrity. The OMC framework fulfills a dual role: (i) it serves as a protective barrier that shields metallic active centers from corrosive species, preventing oxidative dissolution in alkaline environments, and (ii) it provides a robust conductive network for uninterrupted electron flow, significantly enhancing the electrocatalytic properties of the material [17].

Current electrochemical 5-HT sensors employing metal oxide electrode modifiers (CuO [18] and MnFe_2_O_4_ [19]) to enhance sensitivity often suffer from low conductivity and weak catalytic activity compared with those using noble metals, leading to unsatisfactory performance. Although noble metal composites such as Au and Pd offer enhanced catalytic capabilities, their high cost restricts their broad applicability; consequently, the focus has shifted toward developing cost-effective nanomaterials that, even without noble metal catalysts, provide sufficient active sites and high catalytic activity to achieve superior 5-HT detection performance [20]. From this perspective, metal oxides based on copper (Cu) and nickel (Ni) are considered low-cost alternatives to metals such as platinum and ruthenium. This affordability arises from the high natural abundance and simple large-scale processing of both Cu and Ni. In an alkaline environment (pH 8.0), NiO functions as the primary electrocatalytic center by generating active NiOOH species, whereas CuO modulates the electronic environment of the Ni sites to lower the onset potential of the crucial Ni^2+^/Ni^3+^ transition. This synergistic bimetallic interaction is further enhanced by the OMC framework, which ensures high dispersion of active sites and facilitates rapid charge transfer, collectively optimizing the catalytic efficiency of the nanocomposite.

Metal electrocatalysts integrated with OMC exhibit electronic, optical, and magnetic properties that differ significantly from those of their bulk counterparts. Compared to single-metal systems, bimetallic heteroatom catalysts loaded onto OMC create a synergistic environment that enhances electrocatalytic activity and biocompatibility [21,22]. Following this design principle, we report a bimetal-oxide-loaded OMC, namely, CuO–NiO–OMC, which provides enhanced catalytic activity and long-term stability for sensing applications. A disposable electrochemical sensor (screen-printed carbon electrode, SPCE) modified with CuO–NiO–OMC was fabricated for rapid and sensitive detection of 5-HT. CuO–NiO–OMC-modified SPCEs promote selective detection of 5-HT among DA, EP, NE, AA, other biomolecules, and inorganic acids, with a detection limit of 42.5 nM. The integration of CuO and NiO significantly enhances electrochemical performance through electronic synergy at the oxide–oxide interfaces. This synergistic effect is multifaceted: the presence of Cu and Ni modulates electron transfer properties to lower the overpotential for 5-HT oxidation while simultaneously generating a higher density of hydroxylated surface sites that facilitate target adsorption. The ordered mesoporous structure provides high surface area for catalyst dispersion and facilitates physical trapping of 5-HT analyte molecules before electro-oxidation. Thus, the development of NiO–CuO-supported OMC presents a promising approach for 5-HT detection in biological samples.

## 2. Materials and Methods

### 2.1. Materials

Triblock copolymer Pluronic (P123, molecular weight = 5800, EO_20_PO_70_EO_20_), tetraethoxysilane (TEOS, 98%), sucrose, copper(II) acetate, nickel(II) acetate, HCl (37%), H_2_SO_4_ (18.4 M), NaOH, ethanol (99.5%), potassium ferri/ferrocyanide [Fe(CN)_6_]^3−^/^4−^, potassium chloride (KCl), 5–HT, dopamine (DA), epinephrine (EP), norepinephrine (NE), ascorbic acid (AA), tyrosine (Ty), glutamate, glutamine, L-cysteine, lactate, sucrose, NaCl, KCl, and caffeic acid were obtained from Sigma–Aldrich (Seoul, Republic of Korea).

### 2.2. Instrumentation and Measurements

Details of the equipment and measurement techniques are reported in Section S1 of the Supplementary Materials.

### 2.3. Synthesis of CuO–NiO–OMC Nanocomposite

#### 2.3.1. Synthesis of OMC, CuO–OMC and NiO–OMC

Initially, the silica template (SBA-15) was prepared using the non-hydrothermal soft-templating method [23], followed by hard templating to form OMC [24]. A mixture of P123 (2.0 g, surfactant template) and tetraethyl orthosilicate (TEOS) (4.2 g, silica source) in 2.0 M HCl solution (60 g in 80 g water) was stirred at 35 °C for 20 h and then aged overnight at 100 °C to precipitate the mesoporous silica template. The as-prepared template was then combined with a solution containing sucrose (1.5 g, carbon precursor) and H_2_SO_4_ (0.17 g, polymerization catalyst) in water (10 g). The suspension was heated sequentially at 100 °C and 160 °C for 6 h each to polymerize sucrose within the silica pores. Following this step, the brown product was subjected to calcination under nitrogen gas flow, first at 550 °C for 5 h and subsequently at 900 °C for 3 h, transforming the sucrose into a mesoporous carbon framework. Finally, the silica template was removed by adding 1.0 M NaOH to the resulting suspension. The final OMCs were collected via centrifugation, washed, dried at 80 °C, and stored.

CuO–OMC was synthesized [25] by impregnating SBA-15 silica with a solution of sucrose and H_2_SO_4_, followed by drying at 100 °C. The partially carbonized intermediate was then impregnated with copper acetate in ethanol, treated by ultrasonication, and subjected to a second impregnation step with sucrose/H_2_SO_4_ solution before redrying to ensure complete polymerization. The resulting powder was calcined at 900 °C under argon gas for 6 h, simultaneously carbonizing the sucrose and decomposing copper acetate into CuO. The SBA-15 silica template was then selectively removed by washing the composite with 1.0 M NaOH solution at 75 °C, yielding the CuO–OMC nanocomposite after filtration and drying. In this procedure, sucrose carbonized and the copper precursor decomposed within the mesochannels of SBA-15 during calcination to form CuO–OMC. NiO–OMC was synthesized following a similar procedure to that used for CuO–OMC, except that copper acetate was replaced with nickel acetate as the Ni source.

#### 2.3.2. Synthesis of CuO–NiO–OMC

**Scheme 1:**
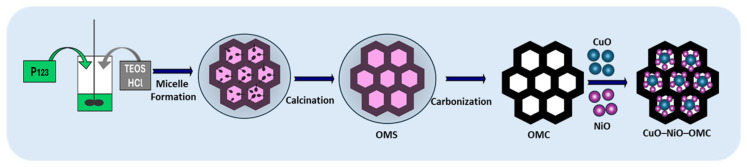
Schematic representation of the synthesis of CuO–NiO–OMC.

CuO–NiO–OMC was synthesized following a procedure similar to that used for CuO–OMC, with the addition of both Cu and Ni precursors. An outline of the synthesis of CuO–NiO–OMC is shown in Scheme 1.

### 2.4. Fabrication of CuO–NiO–OMC/SPCE Sensors

Bare SPCEs were drop-coated with synthesized CuO–NiO–OMC (6.0 µL) and dried at ambient temperature (25 °C). No additional binders were used.

### 2.5. Preparation of Human Serum and Simulated Urine Samples

The performance of the CuO–NiO–OMC/SPCE sensor was assessed using human serum samples (H4522; male human AB plasma) acquired from Sigma–Aldrich (Seoul, Republic of Korea). The human serum samples were diluted 2.0-fold and used for electrochemical analysis. Two distinct simulated urine formulations were prepared according to the protocols established by Laube et al. [26] and Liu et al. [27]. The specific chemical constituents used for the real-sample analyses are detailed in Table S1. The concentrations of 5-HT in the spiked serum and simulated urine samples were calculated using the same method employed for constructing the linear calibration plots.

## 3. Results and Discussion

### 3.1. Physicochemical Characterizations

The structural properties of the synthesized materials (OMC, CuO–OMC, NiO–OMC, and CuO–NiO–OMC) were examined by field-emission scanning electron microscopy (FE–SEM) (Zeiss GEMINI500, Carl Zeiss Microscopy Deutschland GmbH., Oberkochen, Germany), as shown in Figure S1. The OMCs (Figure S1a) exhibited a typical spherical morphology with a slightly smooth surface, while CuO–OMC, NiO–OMC, and CuO–NiO–OMC (Figure S1b–d) exhibited similar spherical morphologies with highly smooth surfaces due to Cu, Ni, and Cu–Ni loading. These results demonstrate that the mesoporous carbon structure was largely preserved, signifying the stability of the carbon framework during metal oxide incorporation and subsequent heat treatment. In CuO–OMC, NiO–OMC, and CuO–NiO–OMC, small brighter spots uniformly dispersed throughout the carbon matrix were observed, evidencing the presence of CuO, NiO, and CuO–NiO.

**Figure 1:**
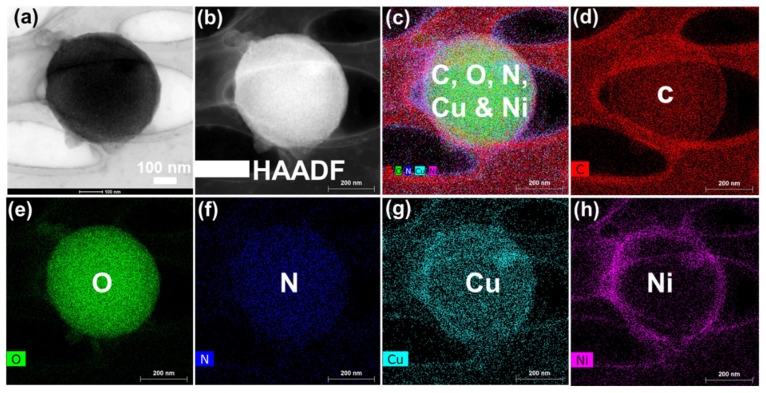
(**a**) TEM image of CuO–NiO–OMC; (**b**) HAADF–STEM image; (**c**) merged HAADF–STEM image showing the distribution of C, O, N, Cu, and Ni; (**d**–**h**) individual elemental maps (scale bar: 200 nm).

Transmission electron microscopy (TEM) images of a single sphere (Figure 1a), along with the high-angle annular dark-field scanning TEM (HAADF–STEM) image (Figure 1b) and its merged image (Figure 1c) of CuO–NiO–OMC, clearly show the material nanostructure, morphology, and elemental distribution, consistent with previous reports [22,28]. The individual elemental maps of CuO–NiO–OMC (Figure 1d–h) illustrate the presence of C, O, N, Cu, and Ni. Numerous Cu- and Ni-containing nanoparticles (NPs) with sizes around 10 nm are uniformly anchored on the OMC spheres. The atomic percentages of C, O, N, Cu, and Ni in CuO–NiO–OMC were 29.23, 65.25, 1.86, 1.66, and 2.04%, respectively (Figure S2). The OMC architecture hosts highly dispersed Cu and Ni oxides with diameters below 10 nm, integrated into the inner and outer mesopore surfaces, ensuring high interfacial contact between the redox-active oxides and the conductive carbon support. The uniform dispersion of CuO–NiO confirms the structural advantage of using OMC as a matrix for supporting these bimetallic oxides. The dominant O content indicates that Cu and Ni exist primarily as metal oxides (CuO–NiO) or mixed Cu/Ni oxides rather than as pure metallic NPs. This spherical OMC matrix combined with the co-localization of active bimetallic oxides is key to the synergistic catalytic effects, yielding an extensive surface area and ample active sites for the adsorption and electro-oxidation of 5-HT [29].

The crystallinity of the materials was confirmed by wide-angle X-ray diffraction (XRD), as shown in Figure S3. The XRD pattern of OMC shows two broad low-intensity humps at ~24° and 43°, corresponding to the (002) and (101) planes of graphitic carbon (JCPDS Card No. 75-1621). The broad feature indicates the amorphous or semi-crystalline structure of the carbon framework [30], which is often masked in the composites by the intense metal/metal oxide crystalline peaks. Upon loading CuO onto OMC, new sharp peaks appeared at approximately 35.5°, 38.7° and 48.7°, corresponding to the (111), (–111), and (–202) planes of CuO, respectively. The twin peak corresponding to the (111) plane matches JCPDS Card 48-1548, confirming the formation of crystalline monoclinic CuO within the carbon matrix [31,32]. The peak centered at 43.3° (inset blue) corresponds to the (111) metallic Cu peak (aligned with JCPDS 04-0836) due to high-temperature calcination at 900 °C under an inert nitrogen atmosphere. The Ni component primarily exists in the cubic Ni (II) oxide phase, aligning with JCPDS Card No. 47-1049. The high-intensity peaks in the NiO–OMC composite were found at 44.5° (111), 51.8° (200), and 76.4° (222), confirming the long-range order of the NiO crystals [33]. The inset provides evidence of partial reduction, where NiO is reduced to its metallic state due to OMC, which acts as a reducing agent during thermal treatment (900 °C) [34,35]. The inset (green) peaks at 44.5° (111) and 51.8° (200) overlap significantly with the NiO peaks. However, it denotes “Metallic Ni,” suggesting that the sharpness and intensity at these positions are a combined contribution of both the oxide (NiO) and metallic (Ni) phases.

The NiO–CuO–OMC material displays a superposition of peaks from OMC, CuO–OMC, and NiO–OMC. Both CuO and NiO peaks are observed, with slight shifts. The (111) peak of NiO and the (–111)/(111) peaks of CuO are clearly visible, confirming that both NiO and CuO phases coexist within the OMC matrix (inset purple). There is a significant overlap between metallic Ni and NiO peaks in these regions. The inset (purple) clarifies that a portion of the Ni existing in its metallic state is likely due to the reducing environment provided by the OMC during synthesis. Additionally, there were many small peaks corresponding to (002), (020), (202), (220), and (–222), signifying highly crystalline oxide phases of CuO and NiO, rather than their metallic clusters. During the synthesis process, the single-metal oxides (CuO–OMC and NiO–OMC) underwent carbothermal reduction to form metallic Cu and Ni; however, the synergistic interaction between the metal species in the CuO–NiO–OMC composite provided a stabilizing effect that preserved the oxide phases, as evidenced by the characteristic (020), (202), (220), and (–222) reflections. The dashed vertical line at 44.5° highlights a distinct shift in the OMC (111) peak, verifying the presence of strong interfacial interactions between the carbon framework and the metal oxides. This structural shift, combined with the preservation of oxide fingerprints, confirms that the bimetallic system possesses a unique chemical environment that resists the full reduction observed in its single-metal counterparts [34,35].

**Figure 2:**
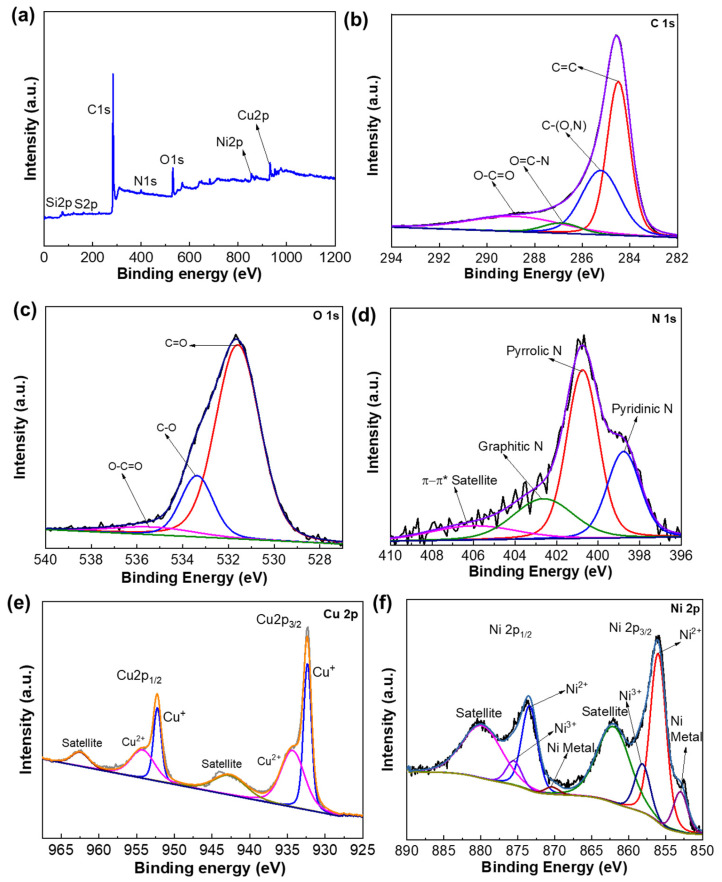
(**a**) XPS survey spectra of the synthesized CuO–NiO–OMC material, (**b**) deconvoluted C 1s region, (**c**) O 1s region, (**d**) N 1s region, (**e**) Cu 2p region, and (**f**) Ni 2p region.

The X-ray photoelectron spectroscopy (XPS) survey spectrum of the CuO–NiO–OMC composite revealed the surface composition and chemical states of the elements present in the material. The survey scan (Figure 2a) confirms the presence of Cu, Ni, O, N, C, Si, and S, indicating successful composite formation. The deconvoluted XPS spectrum of C 1s (Figure 2b) reveals carbon atoms in different chemical states. The main peak observed at a binding energy of 284.48 eV corresponds to sp^2^-hybridized carbon (C=C) bonds, representing graphitic carbon and forming the dominant component of the spectrum. The second peak at 286.22 eV corresponds to C–O/C–N species, typically arising from hydroxyl or epoxy groups on the carbon surface. The third peak at 286.88 eV corresponds to O=C–N, and the fourth peak at a higher binding energy of 288.8 eV is attributed to oxygen-containing O–C=O functional groups, which often serve as anchoring sites for metal oxides. Overall, the C 1s spectrum confirms that the surface carbon is primarily composed of graphitic carbon, with a smaller contribution from oxidized carbon species [36]. The deconvoluted XPS spectrum of O 1s (Figure 2c) shows three peaks at 531.59, 533.38, and 535.27 eV, corresponding to the carbonyl (C=O), C–O (alkoxide/oxygen vacancies), and carboxyl (O–C=O) species respectively. The dominance of the C=O peak suggests a high concentration of carbonyl groups on the surface. The C-O peak indicates the presence of hydroxyl or epoxy groups. The small O-C=O peak at higher binding energy confirms a minor contribution from carboxylic acid groups, which are often located at the edges of OMC sheets. The deconvoluted XPS spectrum of N 1s (Figure 2d) shows four peaks at 398.77 eV (pyridinic N), 400.75 eV (pyrrolic N), 402.64 eV (graphitic N), and 406.0 eV (satellite peak), corresponding to N atoms located at the edge of the carbon lattice, N incorporated into five-membered rings, N substituted into the carbon basal plane, and a weak π-π* satellite peak respectively [37].

The deconvoluted XPS spectrum of the Cu 2p region (Figure 2e) is characterized by spin–orbit doublets and distinct shake-up satellites, confirming the coexistence of Cu^+^ and Cu^2+^ oxidation states in the material. The peaks observed at binding energies of 932.3 eV and 952.4 eV correspond to the Cu 2p_3_/_2_ and Cu 2p_1_/_2_ levels, respectively, associated with Cu^+^ species. Additional peaks at 934.4 eV and 955.0 eV are attributed to the Cu^2+^ 2p_3_/_2_ and Cu^2+^ 2p_1_/_2_ levels, accompanied by strong shake-up features, confirming the presence of divalent Cu. The sharp peak at 932.4 eV is attributed to Cu^+^ (from Cu_2_O), suggesting partial reduction during synthesis, whereas the peak at 934.5 eV is assigned to Cu^2+^ from CuO. Prominent shake-up satellites at ~943 eV and ~962 eV are electronic signatures of the d^9^ configuration, further confirming the Cu^2+^ oxidation state. The spin–orbit splitting between the Cu 2p_3_/_2_ and Cu 2p_1_/_2_ peaks is approximately 20 eV. Overall, the XPS analysis indicates that the material contains both Cu^+^ and Cu^2+^ species, demonstrating a mixed-valence state of Cu resulting from partial oxidation of Cu^+^ to Cu^2+^ [38].

The deconvoluted Ni 2p spectrum (Figure 2f) exhibits a complex multiplet structure, indicating multiple oxidation states. The Ni 2p region shows two primary components: Ni 2p_3_/_2_ (~856 eV) and Ni 2p_1_/_2_ (~874 eV). Peaks at binding energies of ~852.99 and ~870.39 eV correspond to Ni metal peaks. Peaks at binding energies of ~856.39 and ~873.40 eV correspond to Ni^2+^ in the Ni(OH)_2_ or NiO lattice, while the peaks at binding energies of ~858.12 and ~875.52 eV correspond to Ni^3+^ oxidation states associated with oxygen vacancies or the formation of nickel oxyhydroxide (NiOOH) phases. The associated satellite peaks at approximately 879.84 and 862.03 eV correspond to Ni^2+^ species such as NiO or Ni(OH)_2_. These satellite features arise from shake-up processes characteristic of transition metal oxides and are attributed to energy loss related to d–d electronic excitations. The spin–orbit splitting between the Ni 2p_3_/_2_ and Ni 2p_1_/_2_ peaks is approximately 18 eV. Overall, the presence of the Ni metal peak and Ni^3+^ and oxidized Ni^2+^ peaks indicates that the material surface contains Ni in mixed oxidation states, suggesting the coexistence of metallic Ni and NiO phases [39,40]. In summary, the XPS analysis confirms the coexistence of multiple oxidation states of Ni and Cu, along with lattice and surface oxygen species, which collectively support electrocatalysis of the target. The mixed oxidation states of Ni (metal Ni, Ni^2+^, Ni^3+^) and Cu (Cu^2+^, Cu^+^) facilitate efficient charge transfer. The surface-adsorbed oxygen species actively participate in electrocatalysis, and the oxygenated carbon species improve surface adsorption of the target and enhance interfacial charge transfer.

Zeta-potential characterization of the synthesized materials was conducted at pH 7 and 8, and the results are shown in Table 1. Briefly, at pH 7.0 and 8.0, the monometallic oxides, CuO–OMC and NiO–OMC, exhibited moderately negative and slightly positive charges due to partial neutralization of the intrinsic negative surface charge of OMC [41]. At pH 7.0, CuO–NiO–OMC exhibited a slightly positive charge, whereas at pH 8.0, a moderately positive charge was observed. The synergistic combination of CuO and NiO created a distinctly electropositive surface. This electropositive nature lowers the overpotential required to oxidize 5-HT, leading to a stronger and clearer electrical signal compared with OMC or single-metal oxides alone (Table 1).

### 3.2. Electrochemical Characterization of the Sensors in [Fe(CN)_6_]^3−^/^4−^

**Figure 3:**
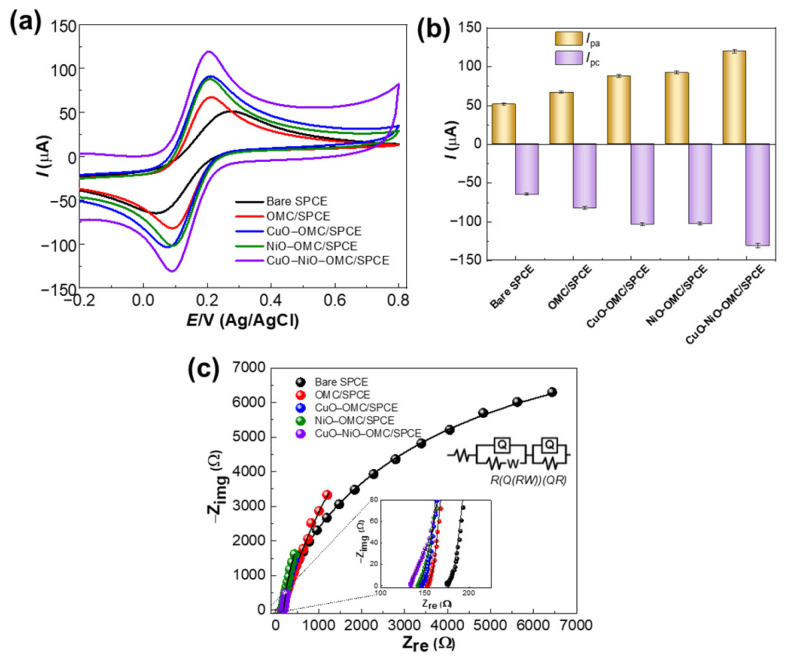
(**a**) CVs of the sensors in 5.0 mM [Fe(CN)_6_]^3−/4−^ at 50 mV s^−1^ scan rate; (**b**) their *I*_pa_ and *I*_pc_ values plotted from (**a**); (**c**) Nyquist plots of the sensors. Inset: The zoomed portion of the plot and the fitted equivalent Randles circuit, *R*(*Q*(*RW*))(*QR*). Error bars: mean ± s.d. (*n* = 3).

Cyclic voltammetry (CV) in [Fe(CN)_6_]^3−/4−^ was performed to reveal the electrochemical characteristics of bare SPCE, OMC/SPCE, CuO–OMC/SPCE, NiO–OMC/SPCE and CuO–NiO–OMC/SPCE sensors in 5.0 mM [Fe(CN)_6_]^3−/4−^ (with 0.1 M KCl) at 50.0 mV s^−1^. Figure 3a shows a pair of redox peaks for all sensors, indicating the quasi-reversible electrochemical behavior of the [Fe(CN)_6_]^3−^/^4−^ couple. On subsequent modification of the SPCE with OMC, CuO–OMC, NiO–OMC and CuO–NiO–OMC, the redox peak currents increase, which is likely due to the number of available metal sites. The CuO–NiO–OMC/SPCE sensors exhibited maximum redox peak currents, demonstrating the presence of abundant available sites provided by the bimetallic CuO–NiO NPs. The anodic (*I*_pa_) and cathodic (*I*_pc_) peak current plot is presented in Figure 3b. The *I*_pa_ values for bare SPCE, OMC/SPCE, CuO–OMC/SPCE, NiO–OMC/SPCE and CuO–NiO–OMC/SPCE sensors were 51.27, 66.73, 87.61, 92.02, and 119.61 µA, respectively. The *I*_pc_ values for bare SPCE, OMC/SPCE, CuO–OMC/SPCE, NiO–OMC/SPCE and CuO–NiO–OMC/SPCE sensors were −65.026, −82.68, −103.65, −102.55 and −131.24 µA, respectively. The enhancement in peak currents for CuO–NiO–OMC/SPCE sensors is attributed to the increased density of electroactive sites and the synergistic integration of CuO–NiO NPs. The electroactive surface area (ESA) of the sensors was calculated using the equation [42].

(1) $$I_{p}=0.4463\;n\;FAC\times \sqrt{\frac{nFvD}{RT}}$$

where *I*_p_ is the forward peak current, n is the number of electrons transferred, F is Faraday’s constant (96,485 C/mol), A is the ESA, C is the concentration of the redox probe, D is the diffusion coefficient of the probe, *v* is the scan rate, R is the universal gas constant, and T is the temperature. The estimated ESA value for CuO–NiO–OMC/SPCE was 0.484 cm^2^, whereas that for the bare SPCE was 0.326 cm^2^.

For a quasi-reversible reaction, the exchange current density ($i_{0}$) was calculated using the Nicholson method [43,44]. The kinetic parameter (Ψ) correlates the observed peak separation in CV ($\Delta E_{p}=116$ mV) with the electron transfer rate. Ψ was calculated using the following equation:

(2) $$\Psi =\frac{0.6288+0.0021\;\Delta Ep}{1-0.017\;\Delta Ep}$$

Substituting Δ*E*_p_ = 116 mV yielded a Ψ value of 0.396. The standard rate constant ($k^{0}$) was calculated using the following expression:

(3) $$k^{0}=\;\Psi \frac{\sqrt{\pi DnFv}}{RT}$$

Upon substituting a scan rate of 50 mV s^−1^ and a diffusion coefficient (D) of 7.63 × 10^−6^ cm^2^ s^−1^, the calculated $k^{0}$ value was 2.705 × 10^−3^ cm s^−1^.

(4) $$i_{0}=nFAk^{0}C^{*}$$

where $k^{0}$ is 2.705 × 10^−3^ cm s^−1^ (derived from $\Delta E_{p}$), the electrode area (A) is 0.1256 cm^2^, the concentration ($C^{*}$) is 5 × 10^−6^ mol cm^−3^, n=1, and F=96,485 C mol^−1^. Substitution of these values yielded an $i_{0}$ value of 163.9 µA. The $i_{0}$ value of 163.9 µA for the CuO–NiO–OMC nanocomposite indicates a high rate of electron transfer at the electrode surface. Compared with typical single-metal-oxide-modified electrodes, which often exhibit values in the range of 10–50 µA, this result demonstrates enhanced catalytic kinetics attributable to the bimetallic synergy and the high surface area of the OMC material.

The electrical properties of the sensors, specifically their resistances, were determined using electrochemical impedance spectroscopy (EIS). The Nyquist plots (Figure 3c) were fitted with a modified Randles circuit, denoted as *R*(*Q*(*RW*))(*QR*). This circuit consists of a solution resistance in series with a parallel combination of a constant phase element (CPE) and a Faradaic branch (charge transfer resistance (*R*_ct_) and Warburg impedance (*W*)). *R*_s_ represents the resistance of the electrolyte solution between the working and reference electrodes. The CPE (double-layer capacitance) represents the electrochemical double layer formed at the interface between the CuO–NiO–OMC surface and the electrolyte. *R*_ct_ is the resistance encountered when electrons move from the redox probe to the electrode (or vice versa) during the redox reaction at the CuO–NiO sites [45]. The OMC increases surface area, providing more space for the double layer and providing a conductive path (decreases *R*_ct_). Since the CuO–NiO acts as an electrocatalyst, the *R*_ct_ semicircle diameter shrinks, signifying better catalytic activity. *W* models the diffusion of the analyte from the bulk solution to the electrode surface. The bare SPCE (black curve) exhibited a significantly large, well-defined semicircle, indicating high interfacial resistance (*R*_ct_ = 312.2 Ω) and a relatively slow electron transfer rate at the unmodified carbon surface. For OMC/SPCE (red curve), the diameter of the semicircle decreased, illustrating a reduced *R*_ct_ (286.8 Ω). This reduction was due to the high surface area and electrical conductivity of OMC, which facilitated faster interfacial electron transfer kinetics. Further decoration with binary composites (CuO–OMC and NiO–OMC) reduced the semicircular arc further and showed a decrease in *R*_ct_ (272.4 Ω and 265.2 Ω). Finally, dual-metal oxide decoration (CuO–NiO–OMC/SPCE) demonstrated the lowest *R*_ct_ value (228.1 Ω), indicating faster interfacial electron transfer. Thus, the EIS results confirm that the synergistic effect between the bimetallic oxides (CuO–NiO) and the OMC framework creates the most efficient pathway for electron transport. Overall, EIS analysis confirms that the CuO–NiO–OMC nanocomposite significantly enhances conductivity and interfacial electron transfer of the sensor, which correlates with the high current responses observed in the CV studies shown in Figure 3a,b. Additionally, the conductivity of the materials was estimated using a four-point probe instrument (DA-SOL Eng Co., Ltd. Hwaseong-si, Gyeonggi-do, Republic of Korea). The conductivities of OMC, CuO–OMC, NiO–OMC, and CuO–NiO–OMC were 0.082, 1.206, 1.200, and 2.242 S cm^−1^, respectively.

The synergistic interaction between CuO and NiO within the bimetallic nanocomposite creates a unique electronic environment characterized by hybridization of Cu and Ni d-orbitals, which significantly enhances electrochemical performance compared with single-metal-oxide systems. This configuration facilitates accelerated redox transitions, such as the conversion of Ni^2+^ to Ni^3+^, by utilizing neighboring Cu species as local electron reservoirs to streamline charge transfer. The efficacy of this interaction is supported by EIS results, where the CuO–NiO–OMC composite exhibits substantially lower *R*_ct_ values than either CuO–OMC or NiO–OMC alone, confirming that bimetallic modification optimizes electronic conductivity and catalytic efficiency. The electronic and structural interplay between Cu and Ni within the OMC framework can be explained as follows: (i) electronic synergy: interaction between CuO and NiO leads to redistribution of electron density at the interface; (ii) charge transfer: electrons shift from one metal species to another (e.g., from Cu to Ni sites), stabilizing higher oxidation states such as Ni^3+^ that are more catalytically active; (iii) improved conductivity: although OMC provides a conductive backbone, the bimetallic interface reduces internal resistance of the catalyst; (iv) lower overpotential: the synergistic effect results in a lower onset potential for redox reactions compared with single NiO or CuO; (v) accelerated kinetics: the presence of Cu facilitates faster electron transport to Ni active sites, thereby increasing $i_{0}$ [34,46]. Thus, the Cu–Ni interaction demonstrates improved electrochemical performance compared with single-metal modification.

### 3.3. Electrochemical Characterization of the Sensors in 5-HT

**Figure 4:**
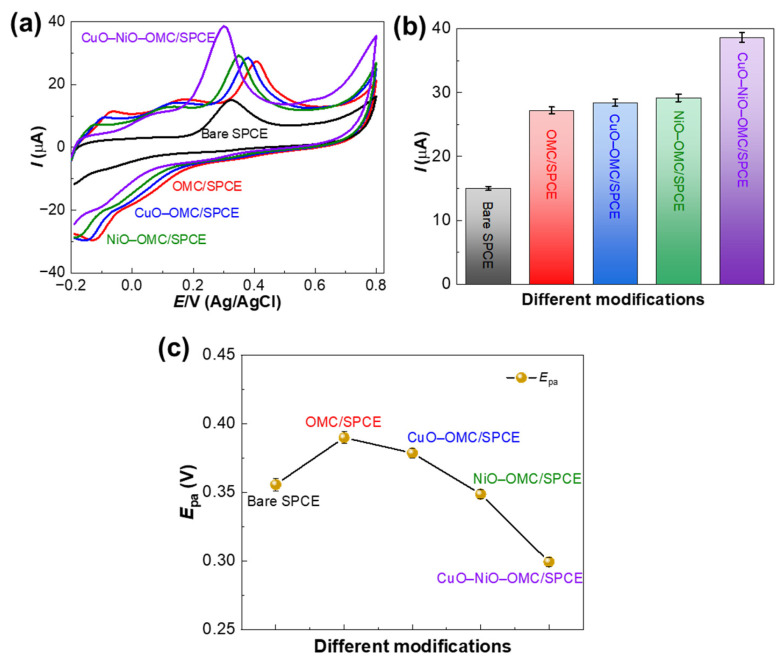
(**a**) CVs of the modified sensors in 0.1 M phosphate buffer (pH 8.0) with 50.0 µM 5-HT; (**b**) corresponding *I*_pa_ value plot; (**c**) *E*_pa_ value plot. Error bars: mean ± s.d. (*n* = 3).

The oxidation of 5-HT at bare SPCE, OMC/SPCE, CuO–OMC/SPCE, NiO–OMC/SPCE, and CuO–NiO–OMC/SPCE sensors was explored using CV scans. Figure 4a presents the CV curves of the sensors in 50.0 µM 5-HT in phosphate-buffered solution (pH 8.0). A small irreversible oxidation peak for 5-HT was observed at the bare SPCE, whereas prominent irreversible oxidation peaks were recorded for OMC/SPCE, CuO–OMC/SPCE, NiO–OMC/SPCE, and CuO–NiO–OMC/SPCE sensors. CuO–NiO–OMC/SPCE showed a maximum *I*_pa_ value of 38.60 μA (2.5-fold compared to the bare SPCE), while the OMC/SPCE, CuO–OMC/SPCE, and NiO–OMC/SPCE indicated *I*_pa_ values of 27.22, 28.41, and 29.17 μA (1.8-fold higher than the bare SPCE) respectively (Figure 4b). The electrochemical performance is primarily driven by the NiO component, which facilitates the Ni^2+^/Ni^3+^ redox couple at the utilized alkaline pH [34]. As shown in Figure 4b, the characteristic *I*_pa_ corresponds to the formation of CuO and NiO–OH, which are the active species promoting 5-HT electro-oxidation. Although NiO provides the catalytic centers, its integration with CuO and the OMC framework is critical; CuO induces a synergistic electronic shift (evidenced by the Ni 2p XPS shift in Figure 2) that lowers the activation energy for this redox transition [47]. The OMC provides a high-surface-area conductive matrix that compensates for the inherent limitations of single-metal oxide NiO alone.

CuO–NiO distributed within the OMC framework serves as high-density active sites that facilitate pre-adsorption of 5-HT, leading to a significant increase in 5-HT flux and subsequent electro-oxidation, resulting in a 2.5-fold increase in signal response. The marginal shift in *E*_pa_ values toward less positive potentials, from +0.3558 V (bare SPCE) to +0.2994 V (CuO–NiO–OMC/SPCE), is attributed to the increased number of active sites in CuO–NiO–OMC (Figure 4c).

### 3.4. Optimization of Solution pH

**Figure 5:**
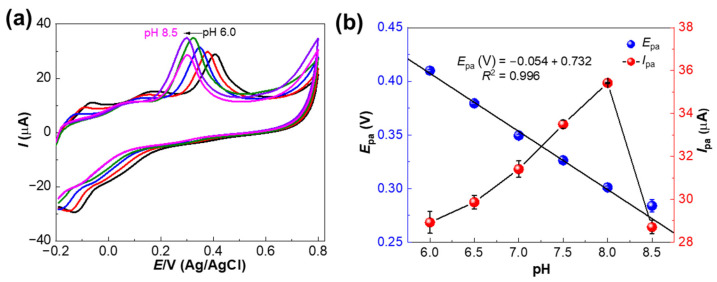
(**a**) CVs of CuO–NiO–OMC/SPCE sensor in 0.1 M phosphate buffer at different pH values (Black: pH 6.0; Red: pH 6.5; Blue: pH 7.0; Green: pH 7.5; Violet: pH 8.0 and Magenta: pH 8.5) with 50.0 µM 5-HT at a scan rate of 50 mV s^−1^; (**b**) corresponding plots of *E*_pa_ and *I*_pa_. Error bars represent mean ± s.d. (*n* = 3).

The effect of buffer pH on 50.0 µM 5-HT detection at the CuO–NiO–OMC/SPCE sensor was evaluated over the pH range of 6.0–8.5, and the results are shown in Figure 5a. As shown in Figure 5a, the sensor exhibited optimal irreversible oxidation performance at pH 8.0, where it achieved the maximum peak current for 5-HT; however, it remained fully functional at pH 7.5, with only a negligible decrease in sensitivity. Above pH 8.0 (pH 8.5), the peak current significantly decreased (Figure 5b), likely due to deprotonation-induced instability of 5-HT or reduced electrode kinetics. Figure 5b explains the linearity between *E*_pa_ and pH with the linear regression *E*_pa_ (V) = −0.054 pH + 0.732 (*R*^2^ = 0.996), suggesting the involvement of an equal number of protons and electrons (*n* = 2). The obtained slope of −54 mV/pH is close to the theoretical Nernstian value of −59 mV/pH, as described by the theoretical equation [9].

Upon increasing the pH from acidic to basic conditions, the *E*_pa_ values shifted linearly toward less positive potentials, likely due to a preceding deprotonation step that facilitates electron transfer. This relationship is better described by the modified Laviron equation for adsorption-controlled irreversible systems [48].

(5) $$Slope=-2.303\left(\frac{mRT}{anF}\right)$$

The experimental slope yields a calculated ratio of protons (*m*) to electrons (*n*) that is approximately equal, indicating that oxidation of 5-HT involves an equivalent transfer of both species. By incorporating the transfer coefficient (α), the universal gas constant (*R*), the absolute temperature (*T*), and the Faraday constant (*F*), the stoichiometric relationship supports the conclusion that 5-HT oxidation proceeds *via* a coupled process involving two electrons and two protons. At alkaline pH 8.0, which is required for 5-HT electro-oxidation, NiO functions as the primary electrocatalytic center through the generation of active NiOOH species, whereas CuO modulates the electronic environment of the Ni sites to lower the onset potential of the Ni^2+^/Ni^3+^ transition [47]. This synergistic bimetallic interaction is further enhanced by the OMC framework, which ensures high dispersion of active sites and facilitates rapid charge transfer, collectively optimizing the catalytic efficiency of the nanocomposite. Overall, stability across the slightly basic range (pH 7.5–8.0) confirms the robust activity of the CuO–NiO–OMC/SPCE sensor; however, pH 8.0 was selected as the standard condition for all experiments to ensure maximum signal intensity.

### 3.5. Effect of Scan Rates on CuO–NiO–OMC/SPCE Sensors

**Figure 6:**
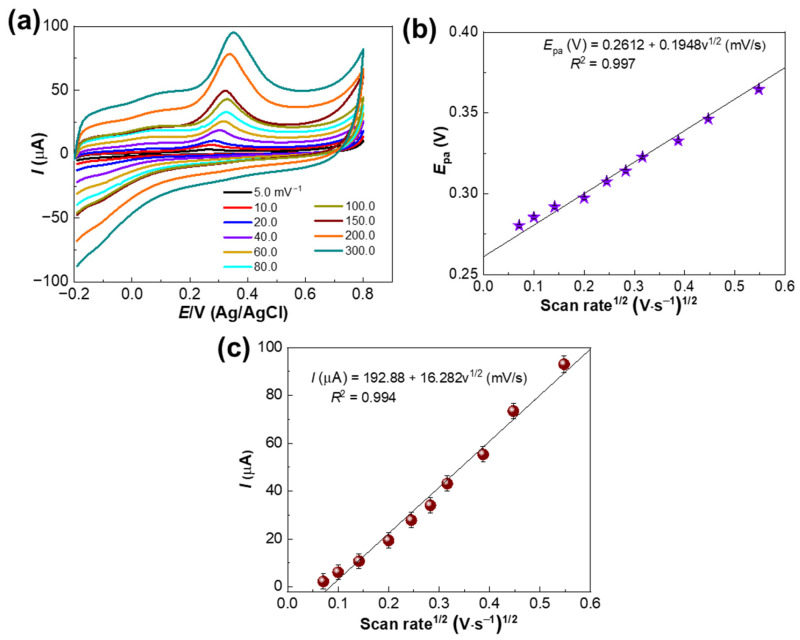
(**a**) CVs of the CuO–NiO–OMC/SPCE sensor in phosphate buffer (pH 8.0) with 50.0 µM 5-HT at scan rates ranging from 5.0 to 300 mV s^−1^; (**b**) corresponding *E*_pa_ plot; (**c**) corresponding *I*_pa_ plot. Error bars represent mean ± s.d. (*n* = 3).

To investigate the kinetic nature of the electrochemical reaction at the CuO–NiO–OMC/SPCE sensor, CV measurements were performed at varying scan rates (*v*) from 5.0 to 300 mV s^−1^ in the presence of 50.0 µM 5-HT. The corresponding voltammograms are shown in Figure 6a.

(i) Peak potential shift: Figure 6b illustrates the relationship between *E*_pa_ and the square root of the scan rate (*v*^1/2^). The *E*_pa_ shifted toward more positive potentials as the scan rate increased, following the linear equation:
  *E*_pa_ = 0.2612 + 0.1948*v*^1/2^ (mV/s), *R*^2^ = 0.997 (6)
  This positive shift is characteristic of an irreversible electrochemical process. In this system, the electron transfer kinetics are not sufficiently fast to maintain Nernstian equilibrium at higher scan rates, resulting in an increased overpotential required to drive 5-HT oxidation.

(ii) Mass transport mechanism: The relationship between *I*_pa_ and *v*^1/2^ was analyzed to determine the mass transport process. As shown in Figure 6c, *I*_pa_ exhibits a strong linear correlation with *v*^1/2^, indicating a diffusion-controlled process.
  *I* (µA) = 192.88 + 16.282*v*^1/2^ (mV/s), *R*^2^ = 0.994 (7)
  This relationship indicates that 5-HT molecules migrate from the bulk solution to the electrode surface via a concentration gradient, which constitutes the rate-determining step of the electrochemical reaction.

(iii) Role of metallic Ni and oxide defects: The presence of metallic Ni^0^ and surface defects (oxygen vacancies) in the CuO–NiO–OMC matrix facilitates the irreversible oxidation of 5-HT. Metallic Ni acts as a conductive bridge, shuttling electrons from the Ni^2+^/Ni^3+^ and Cu^2+^/Cu^3+^ redox-active sites directly to the OMC framework, allowing the sensor to maintain high current responses even at elevated scan rates [47].

### 3.6. Differential Pulse Voltammetry (DPV)

The CuO–NiO–OMC/SPCE sensor was integrated into a portable electrochemical platform and tested using the DPV technique for 5-HT detection. As an electroactive molecule, 5-HT contains a phenolic hydroxyl group (–OH) on its indole ring, which undergoes an irreversible oxidation reaction at the electrode surface during DPV measurements.

**Figure 7:**
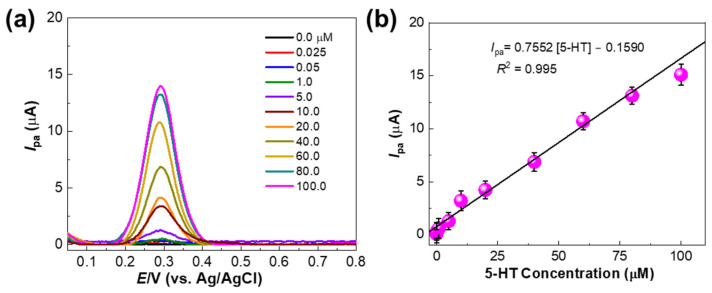
(**a**) DPV responses of CuO–NiO–OMC/SPCE sensor in 0.1 M phosphate buffer (pH 8.0) with different 5-HT concentrations; (**b**) corresponding calibration curve. Error bars represent mean ± s.d. (*n* = 3).

Different concentrations of 5-HT (0.0–80.0 µM) were tested on the CuO–NiO–OMC/SPCE sensor under optimized conditions. The DPV responses were proportional to 5-HT concentration (Figure 7a). The measured responses were plotted to construct a calibration curve (Figure 7b). The linear range was 0.0–80.0 µM, yielding a sensitivity of 1.56 A M^−1^ cm^−2^. The limit of detection (LOD) was calculated using the formula LOD = 3σ/m, where σ is the standard deviation of the replicate blank, and m is the slope of the calibration curve. The calculated LOD was 42.5 nM (S/N = 3). The performance of the sensor is comparable to that of previously reported sensors listed in Table 2, where the LOD of the fabricated CuO–NiO–OMC/SPCE sensor is comparable to those of other reported systems.

### 3.7. Electrochemical Sensing Mechanisms

The primary electro-oxidation of 5-HT is a two-electron and two-proton transfer process. 5-HT undergoes initial oxidation at the electrode surface to form a highly reactive carbocation intermediate, which is subsequently converted into quinoneimine species. The reactive intermediates generated during the process often lead to the formation of dimeric byproducts. These dimers can polymerize on the electrode surface, creating a “poly-serotonin” film that causes electrode fouling [57]. The CuO–NiO–OMC nanocomposite is specifically designed to reduce this fouling by facilitating faster electron transfer and providing a more robust surface chemistry.

**Figure 8:**
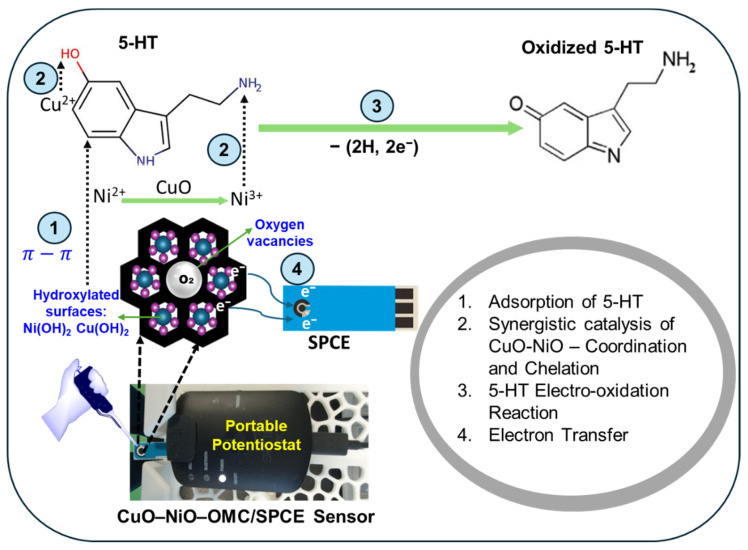
The step-wise electro-oxidation mechanism of 5-HT at the CuO–NiO–OMC/SPCE sensor. Step 1. Electrostatic Attraction or π-π Stacking; Step 2. Coordination and Chelation; Step 3. 5-HT Electro-oxidation Reaction; and Step 4. Electron Transfer.

The electrochemical oxidation mechanism of 5-HT (Figure 8) is described as follows: While OMC provides a high-surface-area conductive framework, the CuO–NiO nanocomposite acts as the catalytic center. CuO and NiO form surface hydroxides, namely, Cu(OH)_2_ and Ni(OH)_2_, at neutral and alkaline pH values; these bimetallic sites likely serve as the primary adsorption interfaces for 5-HT. These hydroxide groups provide hydrogen-bonding sites that facilitate pre-adsorption of 5-HT, which contains both –OH and –NH_2_ groups. 5-HT adsorbs onto the CuO–NiO surface hydroxides. Because the adsorption site is also the electrocatalytic site, electron transfer is rapid. Furthermore, considering the substantial oxide loading, 5-HT molecules are more likely to interact with the accessible CuO–NiO surface than with the underlying, partially shielded OMC layer.

1. Electrostatic Attraction or π-π Stacking

At pH 7.0–8.0, 5-HT molecules are primarily protonated. The –NH_2_ group gains a hydrogen ion to form –NH_3_^+^. Although both the CuO–NiO–OMC surface and the protonated amine are positively charged, the synergistic metal oxide sites act as coordination centers. The combination of CuO and NiO creates localized regions of electron density and electron deficiency, enabling interaction with the electron-rich indole ring of the 5-HT molecule.

2. Coordination and Chelation

The synergy between CuO and NiO provides active metallic sites. NiO interaction: Ni exhibits affinity for nitrogen-containing groups and can form coordinated covalent interactions with the amino groups of 5-HT. CuO interaction: CuO interacts with phenolic –OH groups on the 5-HT ring through hydrogen bonding or metal–oxygen coordination.

3. 5-HT Electro-oxidation Reaction

Upon applying anodic potential, 5-HT diffuses to the CuO–NiO–OMC surface, where it undergoes oxidation to its more stable quinoneimine. Due to strong electron cloud delocalization in the 5-HT molecule, it is very challenging to extract electrons from 5-HT, thus making its electrochemical detection difficult. The developed CuO–NiO–OMC/SPCE sensor can overcome this challenge effectively, since the CuO–NiO–OMC material is rich in oxygen vacancies, which act as the main carriers in promoting electro-oxidation of 5-HT on its surface.

4. Electron Transfer: The Cu and Ni multivalent functional groups also play an important role in adsorbing 5-HT through electrostatic interactions and enable collecting the released electrons and transferring them to the sensor at a faster rate.

### 3.8. Anti-Interference, Reproducibility, and Stability Evaluation

**Figure 9:**
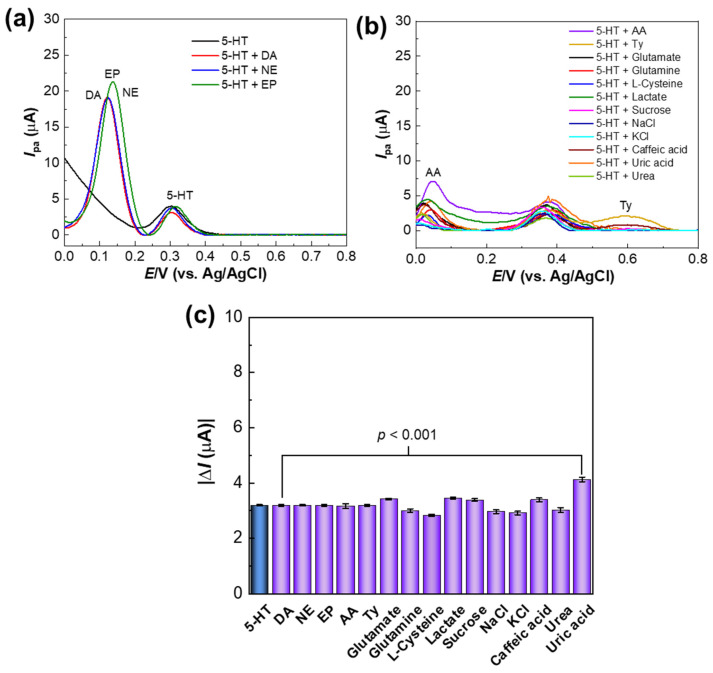
(**a**,**b**) Selectivity of CuO–NiO–OMC/SPCE sensor in 0.1 M phosphate buffer (pH 8.0) with 5-HT in the presence of tested interferents; (**c**) comparison of current responses for 5-HT alone and with interferents.

To evaluate the suitability of the proposed CuO–NiO–OMC/SPCE sensor for selective 5-HT detection, the effects of co-existing interferents were investigated. The sensor response was examined in the presence of 10.0 µM 5-HT and 10-fold higher concentrations (100.0 µM) of other neurotransmitters, including dopamine (DA), norepinephrine (NE), and epinephrine (EP) (Figure 9a). The DPV results show a clearly differentiated peak for 5-HT that was not affected by these neurotransmitters. As shown in Figure 9a, although these catecholamines exhibit significant oxidation peaks at lower potentials (0.12–0.15 V), the oxidation peak for 5-HT remains well resolved at approximately 0.31 V. The wide potential separation (>150 mV) ensures that the 5-HT signal can be accurately quantified without peak overlap, even in the presence of these co-existing neurotransmitters. Additionally, 50-fold higher concentrations (500.0 µM) of common metabolites and ions, including ascorbic acid (AA), tyrosine (Ty), glutamate, glutamine, L-cysteine, lactate, sucrose, caffeic acid, uric acid, urea, NaCl, and KCl, showed no significant interference, enabling 5-HT detection in complex matrices without pre-separation (Figure 9b). Most of these species (e.g., urea and glucose) exhibit negligible electrochemical activity within the target potential window, whereas ascorbic acid shows a peak at a low potential (~0.05 V) and tyrosine appears at a much higher potential (~0.6 V); neither interferes with the characteristic 5-HT peak at 0.31 V.

The bar chart in Figure 9c summarizes the peak current for 5-HT alone compared with 5-HT in the presence of each tested interferent. The current response for 5-HT remained stable, with most interferents causing a variation of less than 5.0%. Although a slight increase was observed in the presence of uric acid, the overall statistical analysis (*p* < 0.001) indicates that the sensor maintained consistent performance across the tested conditions. These results confirm that the CuO–NiO–OMC material provides a selective sensing platform, effectively minimizing interference from common biological species and supporting real-sample analysis in complex matrices.

When integrated into a portable platform, the sensor demonstrated excellent reproducibility and stability for 5-HT sensing. Reproducibility among independently prepared sensors was robust, as five different sensors yielded a low relative standard deviation (RSD) of 1.28% across five repeated measurements, indicating high selectivity, stability, and reproducibility of the developed sensor (Figure S4a,b). Furthermore, stability testing conducted after 0, 1, 2, 3, and 4 weeks showed only minimal variation in signal (below 7%) in the measured current responses, confirming stable performance over time (Figure S4c).

### 3.9. Significance of This Work

The CV and DPV characteristics of the CuO–NiO–OMC/SPCE were compared with those of other contemporary metal oxide sensors for 5-HT sensing. The CuO–NiO–OMC/SPCE sensor is beneficial due to the synergistic effect of bimetallic oxides and the high surface area of OMC, which typically yields higher current densities and lower overpotentials compared to other metal oxide sensors. The table below (Table 3) presents a comparison of our work with the recent literature.

By integrating both CuO and NiO within the OMC composite, this dual-oxide system generates a higher density of oxygen vacancies and synergistic active sites (Ni^2+^/Ni^3+^ and Cu^+^/Cu^2+^) compared to the single-metal-oxide or similar sensors listed in the table, facilitating the achievement of a low LOD.

1. CV Curves—Electrocatalytic activity

The oxidation potential of 5-HT is an indicator of the sensor’s catalytic efficiency. The *E*_pa_ shift in this work (~0.35 V) shows the lowest overpotential among the compared metal oxide platforms (with the complex NiWO_4_/CQD system) [60]. Compared to ZnO–Cu MOF/NF (0.45 V) [59] and CuO/NiO/MWCNT (0.39 V) [58], this sensor has reduced the energy barrier for 5-HT oxidation by 40–100 mV. This negative shift suggests that the OMC provides a more efficient electron transfer pathway than traditional MWCNTs. The synergistic CuO–NiO interface provides highly active sites that trigger oxidation at lower energies. Additionally, in the CV analysis at different scan rates, a linear relationship between the *v*^1/2^ and the *I*_pa_ confirms that the process on CuO–NiO–OMC/SPCE is diffusion-controlled, similar to the transition metal oxide sensors reported in the above table [58,59,60,61].

2. DPV Curves—Sensitivity and LOD

The LOD of the sensor in this work is 42.5 nM. The achieved LOD of 42.5 nM is nearly 15 times lower than that of the similar bimetallic system CuO/NiO/MWCNT (650 nM) [58]. This is likely due to the superior surface area and pore connectivity of OMC compared to the “entangled” network of MWCNTs, which can limit analyte diffusion. While the paper-based Ni(OH)_2_ sensor (22 nM) has a lower LOD, it often lacks the structural robustness of an SPCE [61]. Thus, this work offers a balance of high sensitivity (42.5 nM) with the standardized, reproducible architecture of screen-printed electrodes.

3. Structural synergy: The advantage of OMC

The significance of OMC over MWCNTs or CQDs is evident in the electrochemical results. (i) Mass Transport: The ordered channels in OMC (unlike the random distribution in MWCNTs) allow 5-HT molecules to reach the internal CuO–NiO active sites rapidly. This is reflected in the sharper CV peaks and lower LOD. (ii) Conductivity: The highly graphitic nature of OMC facilitates a faster heterogeneous electron transfer rate (k^0^), which directly translates to the high sensitivity observed in the DPV results. In summary, the sensor in this work has achieved a lower overpotential than the ZnO–Cu MOF [59] and CuO/NiO/MWCNT [58].

Thus, it is evident that the CuO–NiO–OMC nanocomposite created a “bridge” between high-surface-area carbon and bimetallic catalytic centers that is more effective than MWCNTs or MOF-based materials.

### 3.10. Analysis of Real Samples

The sensor demonstrated strong potential for clinical application by successfully quantifying 5-HT in spiked human serum and simulated urine samples. After simple dilution (1:1 in phosphate buffer) without further sample pretreatment, the sensor produced accurate results across various 5-HT concentrations. The average values obtained from triplicate measurements yielded recovery rates ranging from 95.0% to 92.9% for spiked human serum and from 98.3% to 91.1% for spiked simulated urine samples, while maintaining high precision, with RSDs consistently below 5.0% (Table 4). These results demonstrate that CuO–NiO–OMC/SPCE is effective for measuring 5-HT in complex matrices that may otherwise contribute to electrode fouling or signal attenuation. The recovery values obtained from the tested real samples provide proof of concept for potential clinical application. Also, the sensor results for unspiked real samples were validated using the HPLC method. The retention time of 5-HT was 3.5 min. As shown in Table 4, the 5-HT concentrations in unspiked human serum and simulated urine samples 1 and 2 were 0.1, 0.15, and 0.16 µM, respectively. The HPLC results and the sensor results for the real samples were comparable, indicating that the developed sensor is suitable for determination of 5-HT in biological sample matrices.

## 4. Conclusions

In summary, a highly efficient and portable electrochemical sensor, CuO–NiO–OMC/SPCE, for 5-HT detection was successfully developed and characterized. The superior performance of the sensor arises from the structural porosity and adsorption properties of the CuO–NiO–OMC material. The ordered mesoporous structure of OMC provides high surface area and serves as a stable matrix, facilitating uniform dispersion and anchoring of bimetallic CuO–NiO NPs. The developed CuO–NiO composite possesses oxygen vacancies and highly active multivalent CuO and NiO catalytic sites, enabling efficient 5-HT electro-oxidation at the sensor, with a detection limit of 42.5 nM. The sensor also exhibited outstanding selectivity in the presence of competing neurochemicals and biomolecules. When integrated into a portable platform, the sensor was evaluated for analysis of 5-HT in human serum samples, achieving recoveries ranging from 91.1% to 98.3%, with relative standard deviations below 4.5%. The sensor results were also validated against the gold-standard HPLC method to prove its accuracy in 5-HT detection. Thus, the favorable interfacial environment of the CuO–NiO–OMC/SPCE sensor provides an effective approach for 5-HT sensing, supporting molecular-level investigation of brain function.

The high catalytic efficiency of the CuO–NiO–OMC framework offers potential for clinical translational applications. Future efforts will focus on miniaturization of the platform onto flexible polyimide substrates for wearable diagnostics. Additionally, addressing biofouling through biocompatible polymer coatings will be essential for extending application from static in vitro samples to real-time in vivo neurotransmitter monitoring in complex biological matrices.

## Figures and Tables

**Table 1:** Comparison of zeta-potential values for CuO–NiO–OMC/SPCE (at pH 7.0 and 8.0) with single-metal oxides and OMC.

Material	Zeta Potential at pH 7.0 (mV)	Zeta Potential at pH 8.0 (mV)
OMC	−86.23 (Strongly Negative)	−81.05 (Strongly Negative)
CuO–OMC	−41.82 (Moderately Negative)	−37.56 (Moderately Negative)
NiO–OMC	9.96 (Slightly Positive)	11.79 (Slightly Positive)
CuO–NiO–OMC	16.54 (Slightly Positive)	25.42 (Moderately Positive)

**Table 2:** Comparison of CuO–NiO–OMC/SPCE sensor with various reported 5-HT sensors.

Electrochemical Sensor	Linearity (µM)	LOD (nM)	Detection Method	Real Samples	Interferents	Reference
Tri-AgNPs-L-Cys-MXene/GCE	0.5–150 μM	80.0	DPV	Human serum	UA, L-Cys, L-Arg, L-Phe, L-Gly, and several ions	[49]
AgNPs–rGO/Pt/GCE	0.0001–100	0.078	DPV	Artificial urine	DA, AA, UA, 5-Hydroxyindoleacetic acid	[50]
rGO-Ag_2_Se/GCE	0.1–15.0	29.6	DPV	Alzheimer patient’s serum	AA, UA, glucose	[51]
Au-Ns-g-C_3_N_4_/GCE	5 × 10^−7^ and 1 × 10^−3^ M	15.1	CV, LSV	Human embryonic kidney cells	Various biomolecules	[52]
PSS/GCE	0.01–1.0	10	DPV	Human serum	DA, NE, EP, cortisol, glutamine, lactate, glucose, AA, and UA	[53]
MXene-SWCNTs/GCE	4 nM–103.2 μM	1.5	CV, CA	Human umbilical vein endothelial cells	AA, DA, UA, and Ty	[54]
Nb_2_CT_x_/PCN	1.0–100	63.24	CV, DPV	Artificial serum	UA, AA, L-Arg, KCl, DA, L-Cys, and NaCl	[55]
RuO_2_NWs–CeO_2_-AuNFs/f-MWCNTs/GO/SPCE	0.5–100	160	DPV	Pharmaceuticals, serum, plasma	AA, DA, inorganic ions	[56]
CuO–NiO–OMC/SPCE	0.0–80.0	42.5	CV, DPV	Human serum and simulated urine	DA, EP, NE, AA, Ty, glutamate, glutamine, l-cysteine, lactate, sucrose, NaCl, KCl, caffeic acid, uric acid, and urea	This work

L-Cys, L-cysteine; GCE, glassy carbon electrode; rGO, reduced graphene oxide; Pt, platinum; Au-Ns, gold nanostars; g-C_3_N_4_, graphitic carbon nitride; PSS, polystyrene sulfonate; SWCNTs, single-wall carbon nanotubes; Nb_2_CT_x_, niobium carbide; PCN, protonated carbon nitride; RuO_2_NWs, ruthenium oxide nanowires; CeO_2_-AuNFs, cerium oxide gold nanowires; f-MWCNTs, functionalized multiwalled carbon nanotubes; GO, graphite oxide; L-Cys, L-cysteine; L-Arg, L-arginine; L-Phe, L-phenylalanine; L-Gly, L-glycine; Ty, tyrosine.

**Table 3:** Comparison of 5-HT concentration in human serum and simulated urine samples using the DPV technique.

S. No.	Sensor Material and Substrate	Peak Potential (*E*_pa_)	Technique	Linear Range µM	LOD nM	Reference
1	CuO–NiO–OMC/SPCE (This work)	~0.35 V	CV/DPV	0.0–80	42.5	This work
2	CuO/NiO/MWCNT/GCE	~0.39 V	CV/DPV	0.0–50	650	[58]
3	ZnO–Cu MOF/NF	~0.45 V	CV/Amperometry	0.35–135	130	[59]
4	NiWO_4_/CQD/FTO	~0.36 V	CV	0.0–100	134	[60]
5	Ni(OH)_2_/Paper	-	CV	0.01–500	22	[61]

NF, nickel foam; NiWO_4_, nickel tungstate; CQD, carbon quantum dots; FTO, fluorine-doped tin oxide; Ni(OH)_2_, nickel hydroxide.

**Table 4:** Determination of 5-HT in human serum and simulated urine samples using the DPV technique.

Samples	5-HT Added (µM)	5-HT Found (µM) ± S.D	Recovery (%)	RSD (%)	HPLC Found (µM)
Human serum	0.0	0.07	-	1.4	0.1
	1.0	1.01 ± 0.005	95.0	3.4	-
	2.0	1.94 ± 0.007	93.9	0.2	-
	3.0	2.85 ± 0.004	92.9	3.5	-
Simulated urine 1	0.0	0.10	-	1.0	0.15
	1.0	1.08 ± 0.002	98.3	0.9	-
	2.0	1.97 ± 0.007	94.0	1.4	-
	3.0	2.88 ± 0.027	93.1	2.0	-
Simulated urine 2	0.0	0.12	-	0.8	0.16
	1.0	1.08 ± 0.016	97.0	1.1	-
	2.0	1.99 ± 0.012	94.2	0.1	-
	3.0	2.84 ± 0.002	91.1	4.5	-

S.D: standard deviation.

## Data Availability

The original contributions presented in this study are included in the article/Supplementary Material. Further inquiries can be directed to the corresponding authors.

## Supplementary Materials

The following supporting information can be downloaded at https://www.mdpi.com/article/10.3390/bios16040185/s1: Section S1: Instrumentation and measurements. Figure S1: FE–SEM of the synthesized materials; Figure S2: The atomic % of C, O, N, Cu, and Ni elements in CuO–NiO–OMC; Figure S3: Wide angle XRD patterns of the synthesized materials, OMC, CuO–OMC, NiO–OMC, and CuO–NiO–OMC; Figure S4: (a) Reproducibility results of the five CuO–NiO–OMC/SPCE sensors (n = 5) against 5.0 μM 5-HT concentration, (b) Ipa values plot, Error bars: mean ± s.d. (n = 4), (c) the stability of the sensors monitored over 0–4 weeks, shown in relative response %; Table S1: The specific chemical constituents used for synthetic urine preparation.

## Abbreviations

The following abbreviations are used in this manuscript:

AA	Ascorbic acid
AC	Alternating current
Ag/AgCl	Silver/silver chloride reference electrode
Au	Gold
Au-Ns	Gold nanostars
BMJ	*British Medical Journal*
CA	Chronoamperometry
CC	Creative Commons
CE	Capillary electrophoresis
CPE	Constant phase element
Cu	Copper
CuO	Copper(II) oxide
CV	Cyclic voltammetry
DA	Dopamine
DPV	Differential pulse voltammetry
EDX	Energy-dispersive X-ray spectroscopy
EIS	Electrochemical impedance spectroscopy
ELISA	Enzyme-linked immunosorbent assay
EP	Epinephrine
ESA	Electroactive surface area
Fe(CN)_6_^3−^/^4−^	Ferricyanide/ferrocyanide redox couple
FE–SEM	Field-emission scanning electron microscopy
f-MWCNTs	Functionalized multiwalled carbon nanotubes
GCE	Glassy carbon electrode
g-C_3_N_4_	Graphitic carbon nitride
GO	Graphite oxide
HAADF–STEM	High-angle annular dark-field scanning transmission electron microscopy
HCl	Hydrochloric acid
H_2_SO_4_	Sulfuric acid
HPLC	High-performance liquid chromatography
HRTEM	High-resolution transmission electron microscopy
KCl	Potassium chloride
LOD	Limit of detection
LSV	Linear sweep voltammetry
MXene	Two-dimensional transition metal carbide/nitride material
NaCl	Sodium chloride
NaOH	Sodium hydroxide
NE	Norepinephrine
Ni	Nickel
NiO	Nickel(II) oxide
NiOOH	Nickel oxyhydroxide
NPs	Nanoparticles
OMC	Ordered mesoporous carbon
PCN	Protonated carbon nitride
PSS	Polystyrene sulfonate
Rct	Charge transfer resistance
RSD	Relative standard deviation
SPCE	Screen-printed carbon electrode
SWCNTs	Single-walled carbon nanotubes
TEOS	Tetraethyl orthosilicate
TEM	Transmission electron microscopy
Ty	Tyrosine
UA	Uric acid
XPS	X-ray photoelectron spectroscopy
XRD	X-ray diffraction
5-HT	5-Hydroxytryptamine (serotonin)
