# Supplementary material for: A Portable and Highly Selective Electrochemical Sensor Based on Copper–Nickel Oxide-Decorated Ordered Mesoporous Carbon for Serotonin Detection
Source: Biosensors (Basel). 2026 Mar 24;16(4):185. doi: 10.3390/bios16040185 (PMC13114028; doi:10.3390/bios16040185)
Supplement: Supplementary file 1 [file biosensors-16-00185-s001.zip › biosensors-4128699-supplementary.pdf]

Supplementary material

A Portable and Highly Selective Electrochemical Sensor Based on Copper–Nickel Oxide-Decorated Ordered Mesoporous Carbon for Serotonin Detection

Thenmozhi Rajarathinam[1,2]*, Sivaguru Jayaraman[2], Jang-Hee Yoon[3] and Seung-Cheol Chang[2]*

[1]Engineering Research Center for Color-Modulated Extra-Sensory Perception Technology, Pusan National University, Busan 46241, Republic of Korea; thenmozhi@pusan.ac.kr (T.R.)
[2]Department of Cogno-Mechatronics Engineering, College of Nanoscience and Nanotechnology, Pusan National University, Busan 46241, Republic of Korea; sivaguru@pusan.ac.kr (S.J.)
[3]Yeongnam Regional Center, Korea Basic Science Institute, Busan 46742, Republic of Korea; (J.-H. Y)

*Correspondence: thenmozhi@pusan.ac.kr; s.c.chang@pusan.ac.kr.

S.1. Instrumentation and Measurements

Morphological analyses of the synthesized nanomaterials were performed using field-emission scanning electron microscopy (FE–SEM; Zeiss GEMINI 500, Carl Zeiss Mi-croscopy Deutschland GmbH, Oberkochen, Germany) coupled with energy-dispersive X-ray spectroscopy (EDX). High-resolution transmission electron microscopy (HRTEM) images were acquired using a HITACHI H–7600 (200 kV) instrument (Hitachi High-Technologies Corp., Tokyo, Japan). The crystallinity of the materials was examined by X-ray diffraction (XRD; PANalytical X'Pert3 Powder) over a 2θ range of 10–80°. Zeta potential measurements were performed using an electrophoretic light-scattering spectrophotometer (ELS-8000, OTSUKA Electronics Co., Ltd., Japan).

Cyclic voltammetry (CV) and differential pulse voltammetry (DPV) were performed using a portable potentiostat (Sensit Smart, PalmSens Technologies B.V., Houten, Netherlands) with PSTrace software (PalmSens BV, Houten, Netherlands) for data acquisition. A smartphone connected *via* Bluetooth was used to observe and record the CV and DPV signals. Electrochemical impedance spectroscopy (EIS) measurements were carried out using a potentiostat (Model 604E; CH Instruments, Inc., Austin, TX, USA). HPLC analysis was performed using a Shimadzu LC-20AD system equipped with a UV–Vis absorption detector (Nucleosil column). The silica column had a length of 150 mm, an internal diameter of 4.6 mm, and a particle size of 5.0 µm with a pore size of 100 Å. Tetrahydrofuran was used as the mobile phase at a flow rate of 0.5 mg^{-1} mL and a temperature of 30 °C. Zeta potential was examined by an electrophoretic light scattering spectrophotometer (ELS-8000, OTSUKA Electronics Co. Ltd., Japan).

For CV measurements in the redox probe, the sensor was inserted into the portable potentiostat, and 60 µL of 0.1 M KCl solution containing 5.0 mM $[Fe(CN)_6]^{3-/4-}$ was dropped onto the electrode surface. The potential was swept from −0.4 or −0.2 V to +0.8 V. For EIS measurements, 60 µL of the same solution was applied to the electrode surface. The EIS measurements were conducted over a frequency range of 100 kHz to 0.1 Hz with an AC amplitude of 5.0 mV and a DC potential of 250 mV. For CV and DPV measurements in 5-HT solutions, 60 µL of phosphate buffer was applied, and the sensor was polarized from −0.1 to +0.80 V. Stock solutions of different 5-HT concentrations were freshly prepared daily. DPV parameters (pulse time, amplitude, potential step, and scan rate) were systematically optimized using 10.0 µM 5-HT. The sensors (n = 4) were evaluated for each parameter, and the optimal conditions were determined based on the mean Ipa responses. The optimized DPV parameters after 10 s equilibration in 5-HT were: potential step, 5.0 mV; pulse amplitude, 50 mV; pulse time, 75 ms; and scan rate, 10 mV s^{-1}. Calibration curves were generated from baseline-corrected currents obtained at increasing 5-HT concentrations, with standard deviations calculated from more than four measurements. Phosphate buffer (0.1 M) was prepared using 18.2 MΩ water. SPCEs (Model No. C11L) were purchased from Metrohm DropSens (Oviedo, Spain).

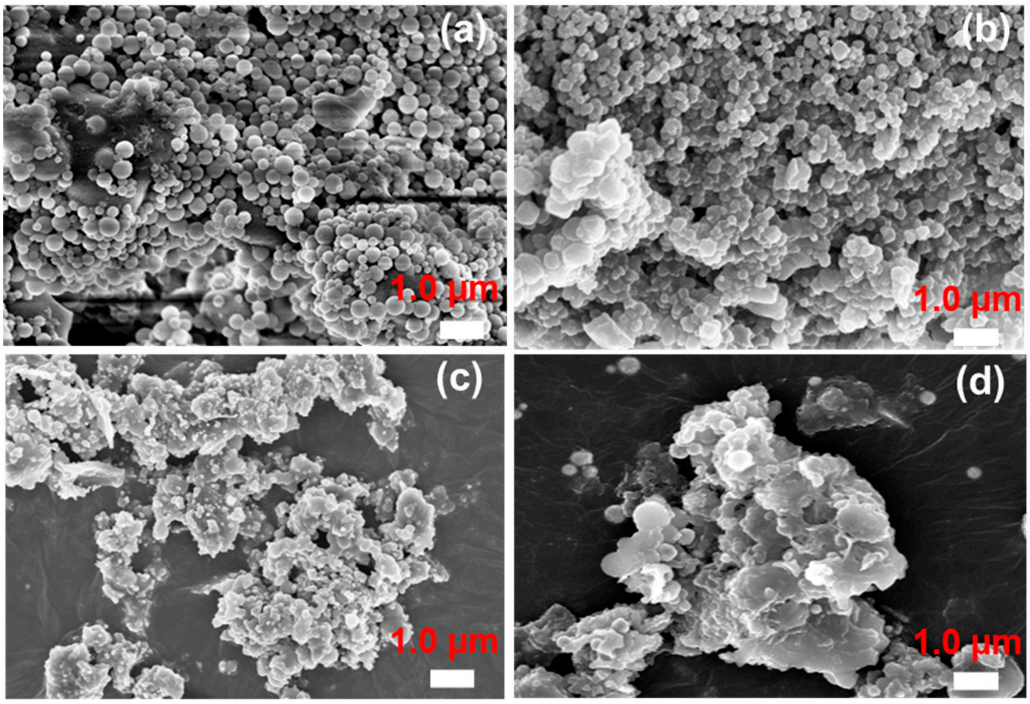

Figure S1. FE–SEM of the synthesized materials (a) OMC, (b) CuO–OMC, (c) NiO–OMC, and (d) CuO–NiO–OMC. Scale bar: 1.0 μm

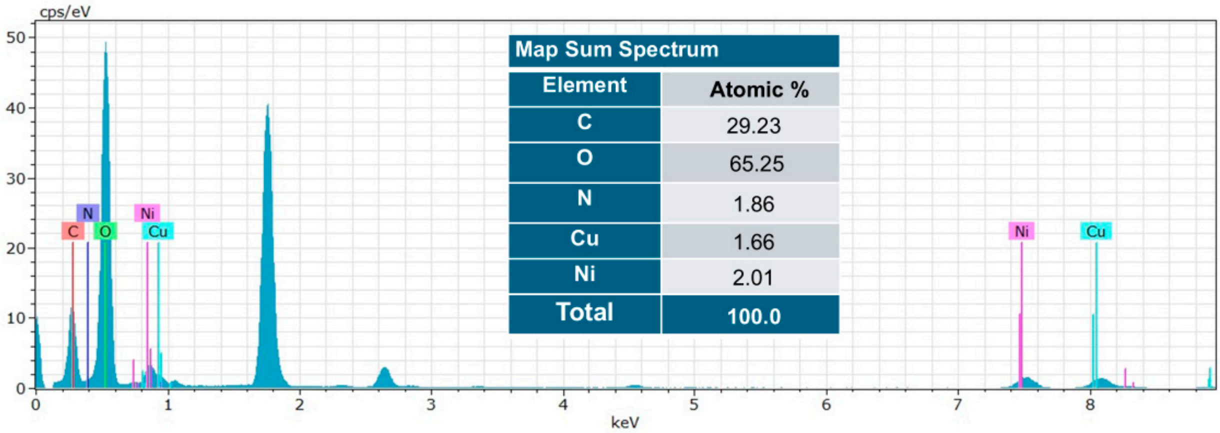

Figure S2. The atomic % of C, O, N, Cu, and Ni elements in CuO–NiO–OMC.

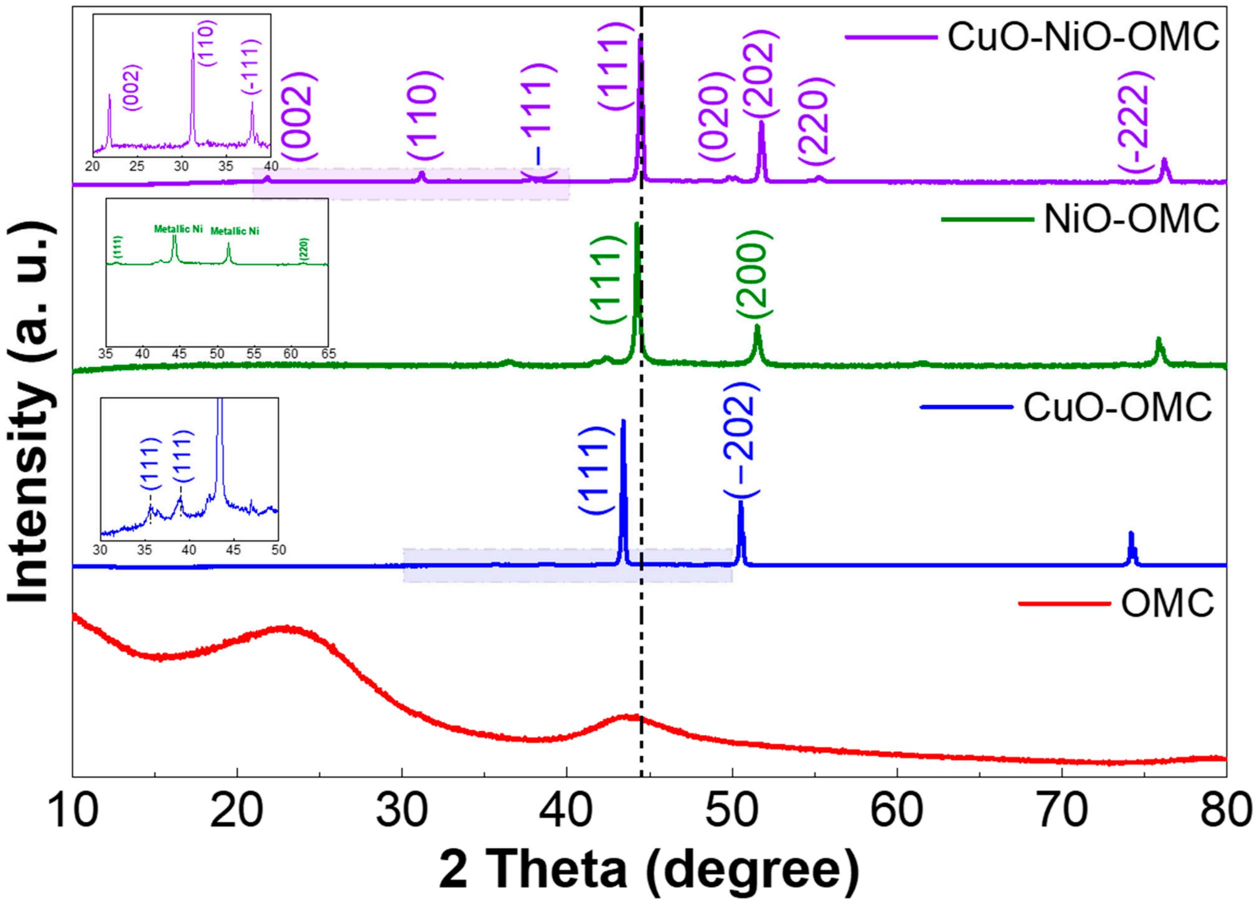

Figure S3. Wide angle XRD patterns of the synthesized materials, OMC, CuO–OMC, NiO–OMC, and CuO–NiO–OMC. The zoomed-in parts of CuO–OMC signifies the presence of metallic Cu (111) peaks and the zoomed-in parts of NiO–OMC indicates metallic Ni (111) peaks. The CuO–NiO–OMC illustrates dominant oxide peaks from both CuO and NiO in addition to metallic Cu and Ni peaks.

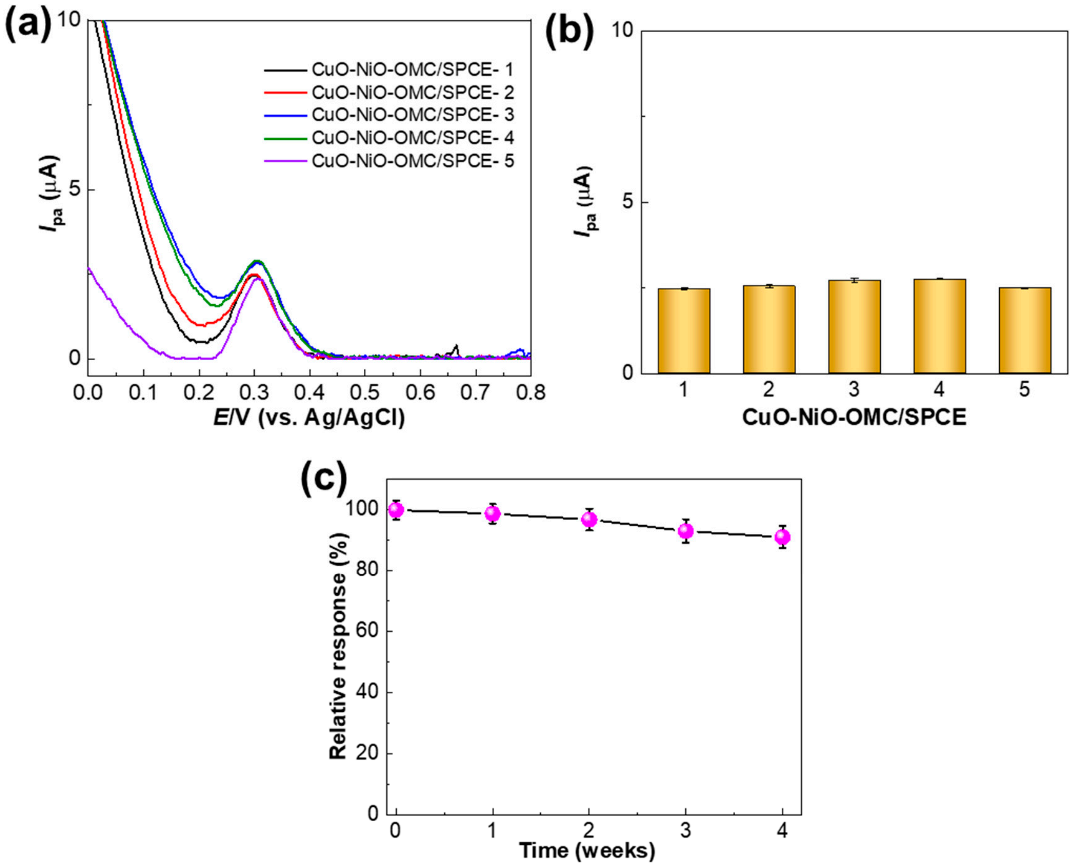

Figure S4. (a) Reproducibility results of the five CuO-NiO-OMC/SPCE sensors (n = 5) against 5.0 μM 5-HT concentration, (b) I_{pa} values plot, Error bars: mean ± s.d. (n = 4), (c) the stability of the sensors monitored over 0-4 weeks, shown in relative response %.

Table S1. The specific chemical constituents used for synthetic urine preparation.

Component	Urea (g L^{-1})	NaCl (g L^{-1})	NH_4Cl (g L^{-1})	Creatinine (g L^{-1})	Na_2HPO_4 (g L^{-1})	KH_2PO_4 (g L^{-1})	Na_2SO_3 (g L^{-1})	$CaCl_2$ (g L^{-1})	KCl (g L^{-1})
Simulated urine - 1	25.0	9.0	3.0	2.0	2.5	2.5	3.0	-	-
Simulated urine - 2	25.0	2.925	1.0	1.1	-	1.4	2.25	1.10	1.6
